# A brave new world: the new normal for general practice after the COVID-19 pandemic

**DOI:** 10.3399/bjgpopen20X101103

**Published:** 2020-06-03

**Authors:** Nada Khan, Daniel Jones, Adam Grice, Sarah Alderson, Stephen Bradley, Paul Carder, Jessica Drinkwater, Helen Edwards, Blessing Essang, Suzanne Richards, Richard Neal

**Affiliations:** 1 Academic Unit of Primary Care, Faculty of Medicine and Health, University of Leeds, Leeds, UK; 2 West Yorkshire Research and Development, NHS Bradford Districts CCG, Bradford, UK

**Keywords:** Family medicine, Community care, Information technology, Primary health care, General practice, COVID-19, coronavirus

## Introduction

General practice in the UK transformed almost overnight in March 2020 in response to the COVID-19 pandemic. Practices largely shut their doors, face-to-face consultations almost exclusively became remote consultations, research evidence was implemented within days of being published, and much routine work postponed and labelled ‘non-essential’. As we settle into this (temporary) new way of working, we have a unique opportunity to reflect on our old and new working practices and decide what we should continue, change, and stop doing. Specifically, we consider what this ‘new normal’ could be in terms of remote consulting, practice re-organisation, use and implementation of evidence, advanced care planning, patient behaviour and chronic disease management, and implications for future practice, research, and policy.

## Remote consulting

The reluctance to introduce, and the limitations of technology to enable, video consultations vanished overnight. Practices introduced a remote ‘total triage’ model in order to protect both patients and the workforce from COVID-19.^1^

Remote consultations currently appear well-received by patients and clinicians, and may improve access to general practice for working age adults, patients with children, those with anxiety or agoraphobia, housebound patients, and those living in remote locations.^2,3^ Mitigating the positives are concerns that remote consultations may increase health inequalities, including for those without access to remote video consulting via smartphones, may disadvantage non-English speakers, and may negatively impact doctor–patient relationships, patient satisfaction, and patient safety.^2,4^ Patients may not disclose some health problems by telephone, including symptoms of serious disease such as cancer. Privacy issues may also make disclosure of domestic violence, safeguarding issues, and mental health problems difficult for vulnerable patients. For older people living alone and palliative care patients, a face-to-face consultation provides a welcome contact mitigating against loneliness; the therapeutic effect of touch should not be forgotten.^5^

The ability to consult remotely has allowed GPs to work from home, undertaking video and telephone consultations alongside administrative work. There is a well-known GP recruitment and retention crisis, with workloads risking GP burnout.^6^ Increased flexibility in working hours and location may address some of these challenges as well as reduce commuting and promote a sustainable work–life balance.^7^ Potential undesirable consequences of remote working include isolation and reduced contact with colleagues and patients. There are also implications for teaching and training in remote consultation skills. In the future, a combination of in-practice and remote working may help some clinicians manage the various demands in general practice and improve work–life balance. Further research is needed to formally assess access, efficiency, unintended consequences, and patient satisfaction with the use of remote consultation systems.

Technology has supported the change towards remote consultations. Eighty per cent of practices are now using AccuRx, a platform that enables SMS, video consulting, and exchanging of documents and images.^8^ Clinical assessment of rashes, throats, and eye complaints are now being done remotely, as well as the sending of patient information leaflets and fit notes. Furthermore, received images (for example, photos) are safely stored in electronic patient records along with an audit trail of electronic contact. These are likely to be enduring changes, reducing the need for patients to visit the practice. The acceptability of telemedicine and mobile applications to remotely manage diabetes and chronic obstructive pulmonary disease have also been previously explored, with promising results.^9,10^ The pandemic has highlighted that there is scope for patients and carers to use medical equipment such as pulse oximeters and blood pressure monitors to assist remote consulting and aid clinical decision-making. Future research should explore whether smartphone devices and apps could do all of this safely, reliably, and accurately in the future.

## Practice reorganisation

During the current pandemic, some primary care networks (PCNs) in England have pooled resources to mitigate potential staffing shortages and develop ‘hot’ and ‘cold’ hubs for reviewing patients.^8^ PCNs, a key component of the NHS Long Term Plan, consist of groups of general practices working together to provide coordinated health care to local populations. Initial reluctance and protests over proposed central PCN contracts still exist, but necessity and clear purpose has resulted in a ‘bottom-up’ collaborative approach which may lead to empowerment of PCNs to work with policymakers over future contracts.^11^

All practices have had to make radical and evolving changes to the way they work and share information with other organisations. In our experience, COVID-19 appears to have increased communication between primary and secondary care to best manage clinical cases. However, co-production of this reorganisation with patients has all but ceased. Patient participation groups and patient experience work were both deemed non-essential work early in the pandemic. The importance and necessity of patient involvement and experiences will need to be taken into account when considering future changes in how we organise general practice.

## Evidence and implementation

Translation of evidence into general practice is unpredictable. It can be a slow and unsystematic process, hindered by the large volume of evidence and research, and by limited time and resources. Evidence and guidelines on COVID-19 management, particularly breathlessness, have evolved rapidly and were introduced into practice at an unprecedented rate, often widely shared between clinicians using social media.^12^ It is possible that clinicians may be more enabled to use guidelines and become more algorithm-driven in the future, resulting in less variation in the quality of care across general practice. As with the Quality and Outcomes Framework, this may have mixed implications. For instance, there is a risk that rapid introduction of new research may have unintended untoward consequences, such as the rapid adoption and then withdrawal of the Roth score to assess breathlessness remotely.^13^

## Advanced care planning

The COVID-19 pandemic has highlighted the importance of advanced care planning, with many patients’ wishes regarding resuscitation and preferred place of care being unknown.^14^ Following the pandemic, advanced care planning will need to sensitively change for the better. General practice is well-placed to have discussions that allow patients to express their wishes, which will reduce unnecessary and possibly undignified hospital admissions. Patients suitable for advanced care planning conversations could be pragmatically and opportunistically identified at the earliest opportunity — perhaps informed by frailty scores — and discussed in multidisciplinary meetings as part of routine care.^15^ The public need to be involved in this decision to address and alleviate fears, and emphasise that these discussions are about providing quality of care.

## Patient behaviour and chronic disease management

During the COVID-19 pandemic so far, appointments in general practice have declined by almost 30%.^16^ In part, this may be because patients are simply not contacting health services due to fear of infection. In parallel, it is possible that patients are managing symptoms through self-care, accessing pharmacists and NHS 111 more readily as a direct response to the government’s messaging about protecting NHS services from becoming overwhelmed. We must be aware of the consequences of this, such as the burden of work for patients and delays in seeking help for potentially life-threatening illnesses. Following the pandemic, changes in patient behaviours and the impact of these on morbidity and mortality will need to be evaluated. The pandemic has led to the suspension of virtually all disease monitoring, health checks, and preventative and screening activity, as these were not deemed ‘essential’. The impact on future provision, value, and acceptance of such services is unknown.

## Conclusion

We are in the midst of the biggest evolving natural experiment in general practice of our lifetimes. Research is already underway to identify the emerging data, challenges, and innovations in general practice amid the COVID-19 pandemic, and we expect this will inform our work and workload in the coming years^17,18^ In the face of rapid change during the COVID-19 pandemic, GPs have an opportunity to permanently change how they consult in terms of video and remote consulting, use of emerging technologies, our dialogue between primary and secondary care, and thinking pragmatically and proactively about end of life care and access to appropriate health care. As a profession we have shown how quickly we can adapt and, going forward, will we now be more open to implementing new technology, systems, and research findings into practice? This article has reflected on the changes occurring and the long-term benefits or harms to patients and practitioners in order to help shape the new ‘normal’ for primary care.

## †

These authors contributed equally to this work
